# New records of amphibians from Bac Kan Province, Vietnam

**DOI:** 10.3897/BDJ.10.e75595

**Published:** 2022-02-08

**Authors:** Anh Mai Luong, Quyen Hanh Do, Chung Van Hoang, Tien Quang Phan, Truong Quang Nguyen, Cuong The Pham

**Affiliations:** 1 Institute of Ecology and Biological Resources, Vietnam Academy of Science and Technology, 18 Hoang Quoc Viet Street, Cau Giay District, Hanoi, Vietnam Institute of Ecology and Biological Resources, Vietnam Academy of Science and Technology, 18 Hoang Quoc Viet Street, Cau Giay District Hanoi Vietnam; 2 Faculty of Environmental Sciences, University of Science, Vietnam National University, 334 Nguyen Trai Street, Thanh Xuan District, Hanoi, Vietnam Faculty of Environmental Sciences, University of Science, Vietnam National University, 334 Nguyen Trai Street, Thanh Xuan District Hanoi Vietnam; 3 Forest Resources and Environment Center, Vinh Quy Street, Thanh Tri District, Hanoi, Vietnam Forest Resources and Environment Center, Vinh Quy Street, Thanh Tri District Hanoi Vietnam; 4 Graduate University of Science and Technology, Vietnam Academy of Science and Technology, 18 Hoang Quoc Viet Street, Cau Giay District, Hanoi, Vietnam Graduate University of Science and Technology, Vietnam Academy of Science and Technology, 18 Hoang Quoc Viet Street, Cau Giay District Hanoi Vietnam

**Keywords:** Nam Xuan Lac, frogs, morphology, taxonomy

## Abstract

**Background:**

Since the establishment of the Nam Xuan Lac Habitat and Species Conservation Area in 2003 in Bac Kan Province, northern Vietnam, only two herpetological studies have been conducted: One recorded 14 species of amphibians from Ban Thi-Xuan Lac area in 2004 and a recent study reported 32 species of amphibians from this protected area in 2019.

**New information:**

As a result of our field surveys in 2020 and 2021, a total of 23 species of amphibians was recorded from the Nam Xuan Lac Habitat and Species Conservation Area. Eight of them are recorded for the first time from Bac Kan Province, comprising one species of Microhylidae, two species of Megophryidae, one species of Dicroglossidae, two species of Ranidae and two species of Rhacophoridae. Besides morphological descriptions, we provide ecological notes of newly-recorded species of amphibians from Bac Kan Province.

## Introduction

The Nam Xuan Lac Habitat and Species Conservation Area (HSCA) was established in 2003 by the People's Committee of Bac Kan. This HSCA encompasses a core zone of 1,788 ha and a buffer zone of 7,508 ha limestone karst forest (Forest Protection Department of Bac Kan Province 2013). In terms of amphibian diversity, Bac Kan Province is one of the most poorly-studied Provinces in northern Vietnam. In their herpetofaunal book of Vietnam, Nguyen et al. (2009) recorded 27 species of amphibians from Bac Kan Province and most of them were reported from Ba Be National Park. Le et al. (2004) recorded 14 species of amphibians from Ban Thi-Xuan Lac Commune. Recently, Tran (2019) reported 32 species of amphibians from Nam Xuan Lac Habitat and Species Conservation Area.

As a result of our recent field surveys in the Nam Xuan Lac Habitat and Species Conservation Area, Bac Kan Province, we herein report eight new records from this Province.

## Materials and methods

### Sampling

**Figure 1.**
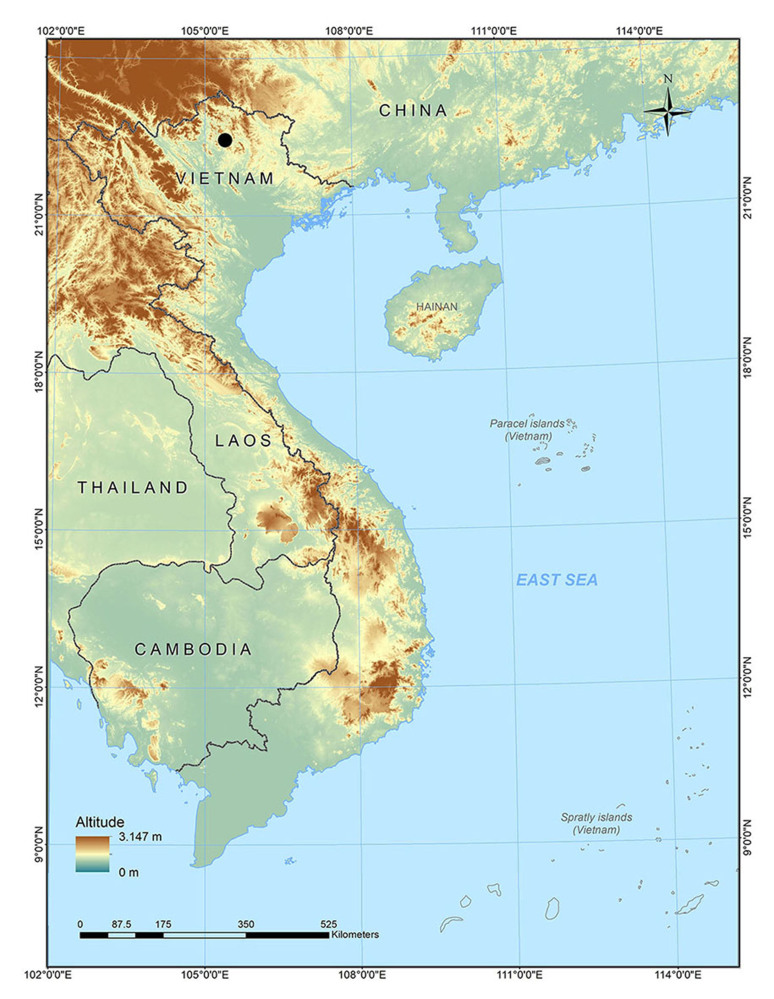
Map showing the Nam Xuan Lac Habitat and Species Conservation Area in Bac Kan Province (black circle), north-eastern Vietnam.

**Figure 2.**
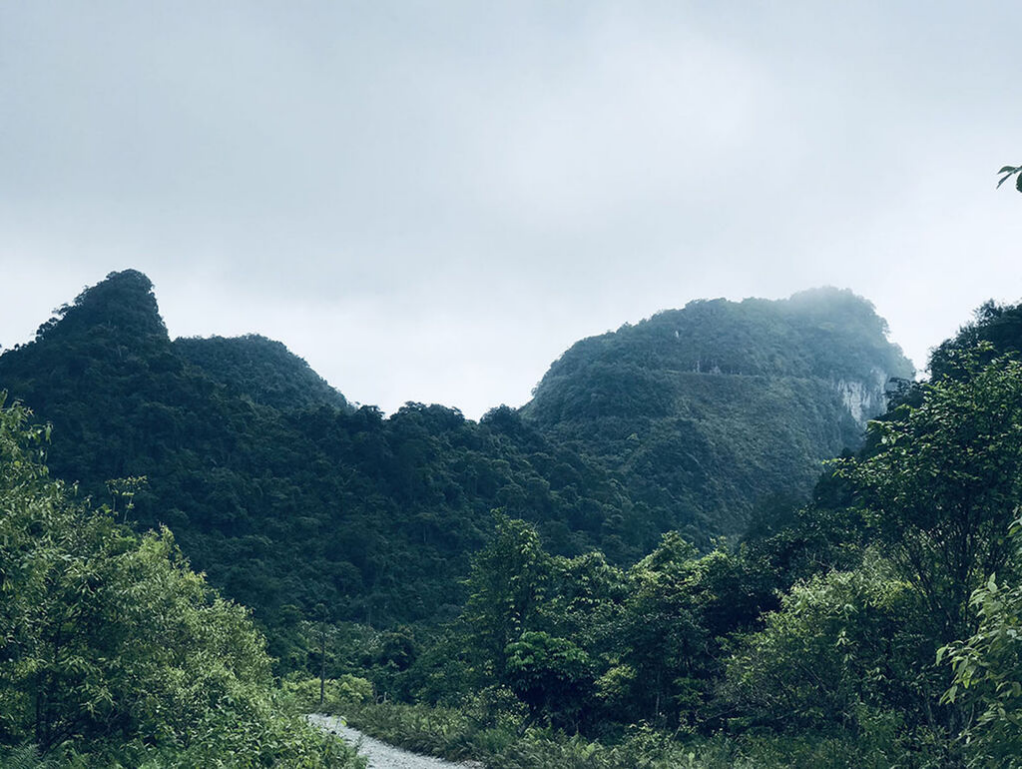
Limestone karst forest of the Nam Xuan Lac Habitat and Species Conservation Area, Bac Kan Province, Vietnam.

**Figure 3.**
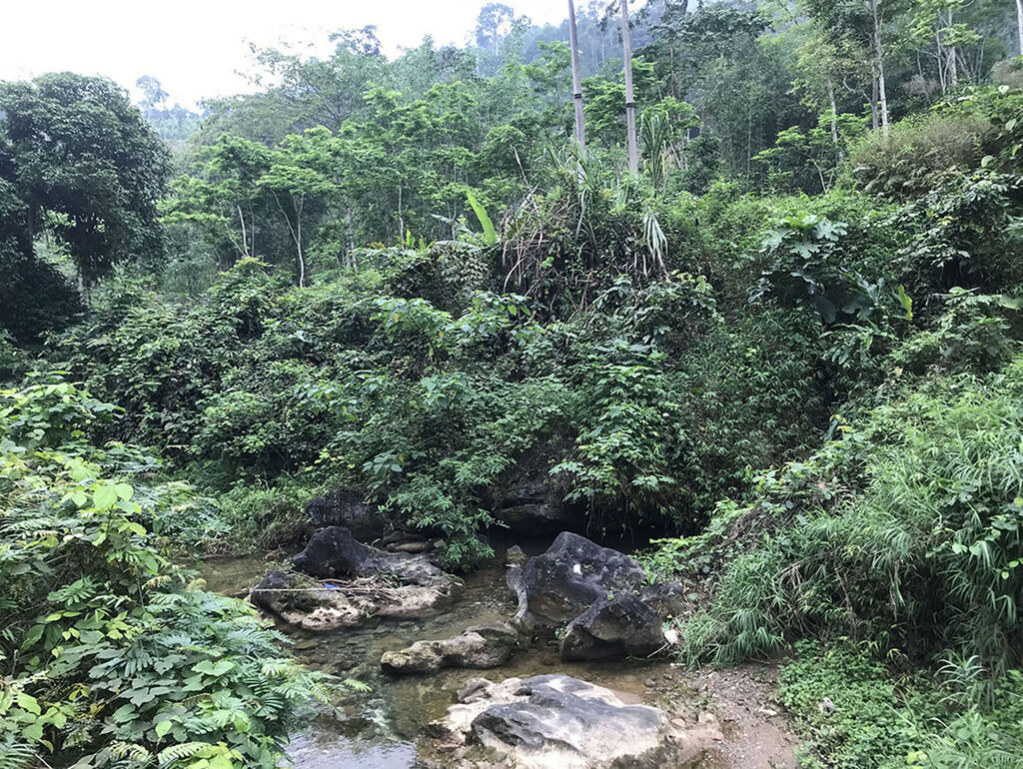
A surveyed stream in the Nam Xuan Lac Habitat and Species Conservation Area, Bac Kan Province, Vietnam.

Field surveys were conducted by Anh Mai Luong, Cuong The Pham, Dung Trung Le, Quyen Hanh Do, Tien Quang Phan and Truong Quang Nguyen (hereafter Luong et al.) from 24 to 29 August 2020 and from 22 to 28 April 2021 in Nam Xuan Lac HSCA, Bac Kan Province (Figs 1, 2, 3). The coordinates (WGS 84) and elevations were determined by using the GPS Garmin 60CX.

Specimens were collected between 19:00 and 24:00 h. After taking photographs in life, specimens were euthanised in a closed vessel with a piece of cotton wool containing ethyl acetate (Simmons 2002), fixed in 80% ethanol for five hours and then later transferred to 70% ethanol for permanent storage. Tissue samples were preserved separately in 70% ethanol prior to fixation. Specimens referred in this paper are deposited in the collection of the Institute of Ecology and Biological Resources (IEBR), Hanoi, Vietnam.

### Morphological examination

Measurements were taken on preserved specimens with a set of digital calipers to the nearest 0.1 mm. The following abbreviations are used: SVL = snout-vent length, HL = head length (measured as a parallel line to the vertebral column from posterior margin of mandible to tip of snout), HW = maximum head width (across angles of jaws), RL = rostral length (from anterior corner of orbit to tip of snout), NS = distance from nostril to the tip of snout, EN = distance from anterior corner of orbit to the nostril, IND = internarial distance, IOD = interorbital distance, ED = eye diameter, UEW = maximum width of upper eyelid, DAE = distance between anterior corners of orbits, MN = posterior margin of mandible to nostril, MFE = posterior margin of mandible to anterior corner of orbit, MBE = posterior margin of mandible to posterior corner of orbit; DPE = distance between posterior corners of orbits, TYD = tympanum diameter, TYE = distance from anterior margin of tympanum to posterior corner of orbit, FLL = forearm length, from elbow to base of outer palmar tubercle, HAL = hand length, from base of outer palmar tubercle to tip of third finger, FL1–4 = Finger length I–IV, OPT = outer palmar tubercle length, IPT = inner palmar tubercle length, NPL = nuptial pad length, FeL = femur length (from vent to knee), TbL= tibia length (from knee to tarsus), TbW = maximum tibia width, FoL = foot length (from tarsus to the tip of fourth toe), TL1–5 = toe length I–V and IMT = inner metatarsal tubercle length. For the webbing formula, we followed Glaw and Vences (2007). Sex was determined by the presence of nuptial pads and based on gonadal inspection.

## Taxon treatments

### Microhyla butleri

Boulenger, 1900

0EF3C61A-F9CD-5B8D-89A6-78F36A66A8D3

#### Materials

- **Type status:** Other material. **Occurrence:** catalogNumber: IEBR A.4877; individualCount: 1; sex: female; lifeStage: adult; **Taxon:** scientificName: *Microhylabutleri*; class: Amphibia; order: Anura; family: Microhylidae; genus: Microhyla; specificEpithet: *butleri*; scientificNameAuthorship: Boulenger, 1900; **Location:** country: Vietnam; countryCode: VN; stateProvince: Bac Kan; locality: Nam Xuan Lac HSCA; verbatimElevation: 699 m; verbatimLatitude: 22°16.470’N; verbatimLongitude: 105°31.337’E; verbatimCoordinateSystem: WGS84; **Event:** eventDate: 28 August 2020; eventRemarks: collected by L. M. Anh and D. H. Quyen; **Record Level:** language: en; collectionCode: Amphibia; basisOfRecord: PreservedSpecimen
- **Type status:** Other material. **Occurrence:** catalogNumber: IEBR A.4878; individualCount: 1; sex: female; lifeStage: adult; **Taxon:** scientificName: *Microhylabutleri*; class: Amphibia; order: Anura; family: Microhylidae; genus: Microhyla; specificEpithet: *butleri*; scientificNameAuthorship: Boulenger, 1900; **Location:** country: Vietnam; countryCode: VN; stateProvince: Bac Kan; locality: Nam Xuan Lac HSCA; verbatimElevation: 321 m; verbatimLatitude: 22°17.130’N; verbatimLongitude: 105°33.428’E; verbatimCoordinateSystem: WGS84; **Event:** eventDate: 27 April 2021; eventRemarks: collected by L. M. Anh and D. H. Quyen; **Record Level:** language: en; collectionCode: Amphibia; basisOfRecord: PreservedSpecimen
- **Type status:** Other material. **Occurrence:** catalogNumber: IEBR A.4879; individualCount: 1; sex: female; lifeStage: adult; **Taxon:** scientificName: *Microhylabutleri*; class: Amphibia; order: Anura; family: Microhylidae; genus: Microhyla; specificEpithet: *butleri*; scientificNameAuthorship: Boulenger, 1900; **Location:** country: Vietnam; countryCode: VN; stateProvince: Bac Kan; locality: Nam Xuan Lac HSCA; verbatimElevation: 321 m; verbatimLatitude: 22°17.130’N; verbatimLongitude: 105°33.428’E; verbatimCoordinateSystem: WGS84; **Event:** eventDate: 27April 2021; eventRemarks: collected by L. M. Anh and D. H. Quyen; **Record Level:** language: en; collectionCode: Amphibia; basisOfRecord: PreservedSpecimen

#### Description

SVL 21.3-24.1 mm; head longer than wide (HL 7.7-8.6 mm, HW 6.5-7.7 mm); snout round, longer than eye diameter (RL 2.6-2.8 mm, ED 2.0-2.2 mm); nostrils round, closer to the tip of snout than to eye (NS 1.1-1.6 mm, EN 1.3-1.6 mm); canthus rostralis indistinct, loreal region oblique, not concave; tympanum indistinct; vomerine teeth absent; tongue notched posteriorly. Forelimbs: Forearm slender (FLL 4.2-5.4 mm), hand length (HAL 8.7-10.4 mm); relative finger lengths I < II < IV < III, tips of fingers pointed; fingers free of webbing. Hind-limbs: Thigh slender (FeL 9.1-12.8 mm); tibia five times longer than wide (TbL 10.5-14.6 mm, TbW 1.9-3.1 mm); relative toe lengths I < II < V < III < IV; webbing formula I1-1½II1-2III2-3IV3-2V; tibio-tarsal articulation reaching to the eye when leg adpressed along body. Skin: Dorsum smooth, but with some rather large smooth flattened pustules on front part of dorsum; supratympanic fold indistinct; throat, chest, belly and underside of limbs smooth; cloacal region granular.

**Figure 4.**
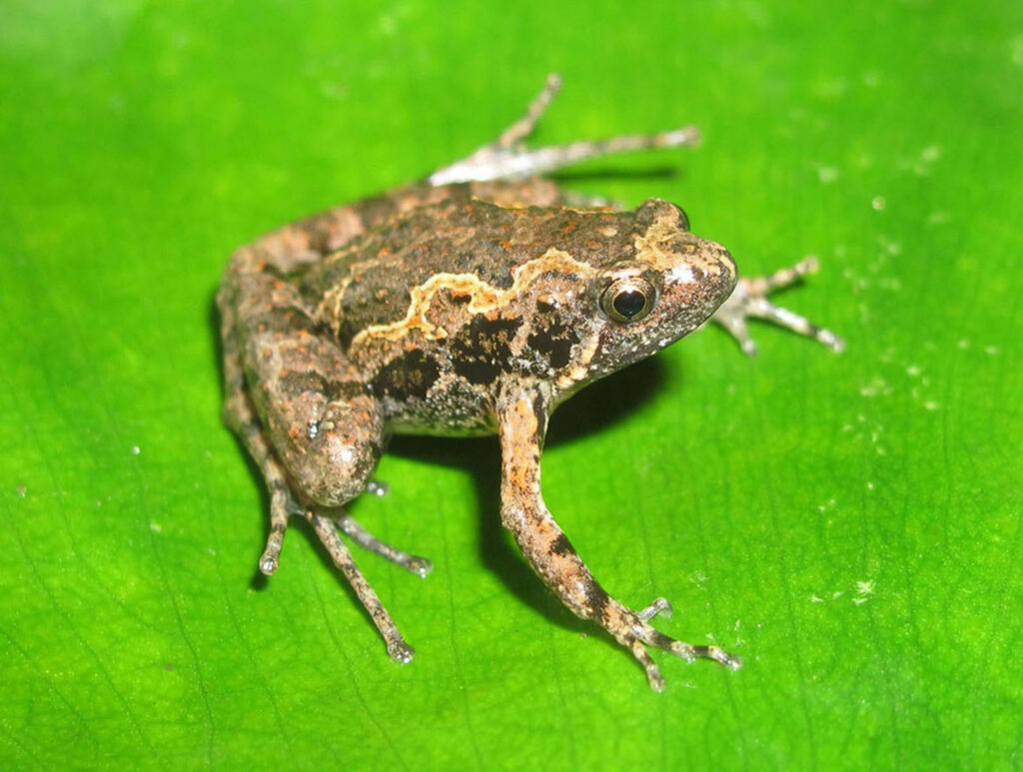
The female specimen of *Microhylabutleri* (IEBR A.4877; SVL 21.3 mm) in life.

Colouration in life: Dorsal surface of head and body grey with brownish and reddish marking, in X-shape; a whitish stripe from eye to anterior shoulder; flank grey with black spots, dorsal surface of limbs grey with dark transverse bars; belly cream; throat and chest mottled with dark brown (Fig. 4) (determination after Bourret (1942), Taylor (1962)).

#### Distribution

This is a common species in Vietnam (Nguyen et al. 2009, Frost 2021). Elsewhere, this species has been recorded from China, Myanmar, Laos, Thailand, Cambodia, Malaysia and Singapore (Nguyen et al. 2009, Frost 2021).

#### Ecology

Specimens were found between 19:30 and 20:30 h on the ground. The surrounding habitat was mixed secondary forest of small hardwoods and shrubs.

### Leptobrachella minima

(Taylor, 1962)

EC4C0495-18D9-525C-A91D-05EBFEAC89BE

#### Materials

- **Type status:** Other material. **Occurrence:** catalogNumber: IEBR A.4880; individualCount: 1; sex: male; lifeStage: adult; **Taxon:** scientificName: *Leptobrachellaminima*; class: Amphibia; order: Anura; family: Megophryidae; genus: Leptobrachella; specificEpithet: *minima*; scientificNameAuthorship: Taylor, 1962; **Location:** country: Vietnam; countryCode: VN; stateProvince: Bac Kan; locality: Nam Xuan Lac HSCA; verbatimElevation: 342 m; verbatimLatitude: 22°15.860’N; verbatimLongitude: 105°29.268’E; verbatimCoordinateSystem: WGS84; **Event:** eventDate: 23April 2021; eventRemarks: collected by L. M. Anh and D. H. Quyen; **Record Level:** language: en; collectionCode: Amphibia; basisOfRecord: PreservedSpecimen
- **Type status:** Other material. **Occurrence:** catalogNumber: IEBR A.4881; individualCount: 1; sex: male; lifeStage: adult; **Taxon:** scientificName: *Leptobrachellaminima*; class: Amphibia; order: Anura; family: Megophryidae; genus: Leptobrachella; specificEpithet: *minima*; scientificNameAuthorship: Taylor, 1962; **Location:** country: Vietnam; countryCode: VN; stateProvince: Bac Kan; locality: Nam Xuan Lac HSCA; verbatimElevation: 864 m; verbatimLatitude: 22°17.260’N; verbatimLongitude: 105°31.138’E; verbatimCoordinateSystem: WGS84; **Event:** eventDate: 24April 2021; eventRemarks: collected by H. V. Chung and P. Q. Tien; **Record Level:** language: en; collectionCode: Amphibia; basisOfRecord: PreservedSpecimen
- **Type status:** Other material. **Occurrence:** catalogNumber: IEBR A.4882; individualCount: 1; sex: male; lifeStage: adult; **Taxon:** scientificName: *Leptobrachellaminima*; class: Amphibia; order: Anura; family: Megophryidae; genus: Leptobrachella; specificEpithet: *minima*; scientificNameAuthorship: Taylor, 1962; **Location:** country: Vietnam; countryCode: VN; stateProvince: Bac Kan; locality: Nam Xuan Lac HSCA; verbatimElevation: 377 m; verbatimLatitude: 22°16.798’N; verbatimLongitude: 105°33.358’E; verbatimCoordinateSystem: WGS84; **Event:** eventDate: 26April 2021; eventRemarks: collected by H. V. Chung and P. Q. Tien; **Record Level:** language: en; collectionCode: Amphibia; basisOfRecord: PreservedSpecimen
- **Type status:** Other material. **Occurrence:** catalogNumber: IEBR A.4883; individualCount: 1; sex: male; lifeStage: adult; **Taxon:** scientificName: *Leptobrachellaminima*; class: Amphibia; order: Anura; family: Megophryidae; genus: Leptobrachella; specificEpithet: *minima*; scientificNameAuthorship: Taylor, 1962; **Location:** country: Vietnam; countryCode: VN; stateProvince: Bac Kan; locality: Nam Xuan Lac HSCA; verbatimElevation: 377 m; verbatimLatitude: 22°16.798’N; verbatimLongitude: 105°33.358’E; verbatimCoordinateSystem: WGS84; **Event:** eventDate: 26April 2021; eventRemarks: collected by H. V. Chung and P. Q. Tien; **Record Level:** language: en; collectionCode: Amphibia; basisOfRecord: PreservedSpecimen
- **Type status:** Other material. **Occurrence:** catalogNumber: IEBR A.4884; individualCount: 1; sex: female; lifeStage: adult; **Taxon:** scientificName: *Leptobrachellaminima*; class: Amphibia; order: Anura; family: Megophryidae; genus: Leptobrachella; specificEpithet: *minima*; scientificNameAuthorship: Taylor, 1962; **Location:** country: Vietnam; countryCode: VN; stateProvince: Bac Kan; locality: Nam Xuan Lac HSCA; verbatimElevation: 723 m; verbatimLatitude: 22°16.450’N; verbatimLongitude: 105°30.712’E; verbatimCoordinateSystem: WGS84; **Event:** eventDate: 25 August 2020; eventRemarks: collected by L. M. Anh, D. H. Quyen, and P. Q. Tien; **Record Level:** language: en; collectionCode: Amphibia; basisOfRecord: PreservedSpecimen

#### Description

Size small (SVL 27.3-30.8 mm in males; SVL 33.9 mm in female); head longer than wide (HL 10.6-11.6 mm, HW 9.3-10.6 mm in males; HL 13.8 mm, HW 12.6 mm in female); snout protruding, longer than eye diameter (RL 4.1-4.5 mm, ED 3.7-4.8 mm in males; RL 5.3 mm, ED 4.9 mm in female); nostrils oval, closer to the tip of snout than to eye (NS 1.7-2.7 mm, EN 2.2-2.7 mm in males; NS 1.9 mm, EN 3.3 mm in female); canthus rostralis distinct, loreal region concave; tympanum round, distinct; vomerine teeth absent; tongue deeped notched. Forelimbs: Forearm rather thin (FLL 6.4-7.0 mm in males; FLL 8.3 mm in female), hand length (HAL 12.6-15.8 mm in males; HAL 19.8 mm in female); relative finger lengths I < II < IV < III, tips of fingers not enlarged; fingers free of webbing. Hind-limbs: Thigh short (FeL 11.8-14.9 mm in males; FeL 16.8 mm in female); tibia five times longer than wide (TbL 13.5-14.7 mm, TbW 2.5-3.0 mm in males; TbL 17.4 mm, TbW 3.1 mm in female); relative toe lengths I < II < III < V < IV; tibio-tarsal articulation reaching to the eye when leg adpressed along body. Skin: Dorsal surface of head smooth; dorsum and upper part of flanks with tubercles and glandular folds; supratympanic fold distinct; dorsolateral fold absent; dorsal surface of limbs with tubercles and glandular folds; ventral surface smooth.

**Figure 5.**
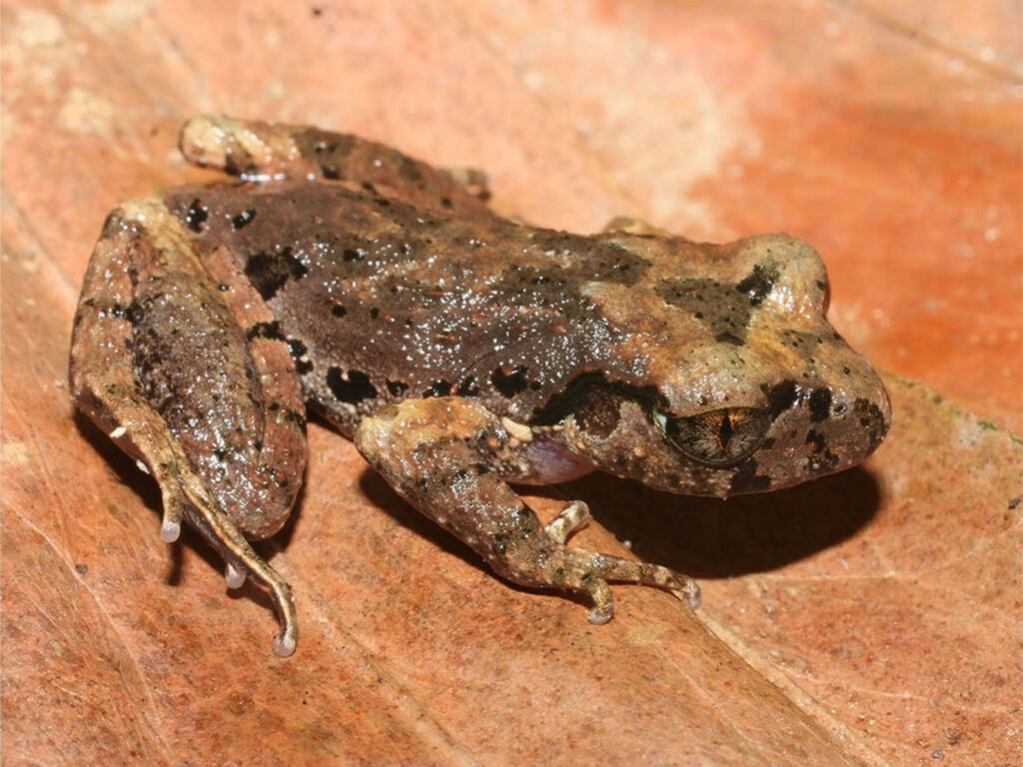
The male specimen of *Leptobrachellaminima* (IEBR A.4880; SVL 30.8 mm) in life.

Colouration in life: Dorsal surface of head and body brown grey with triangular marking between eyes, some grey spots in middle of back; dorsal surface of fore- and hind-limbs brown grey with dark bars; throat and chest transparent grey, border of throat grey with white spots; belly white (Fig. 5) (determination after Taylor (1962), Pham et al. (2016)).

#### Distribution

In Vietnam, *L.minima* was known from Son La, Dien Bien, Hoa Binh, Quang Ninh and Thanh Hoa Provinces (Frost 2021). Elsewhere, this species has been recorded from Thailand and Laos (Frost 2021).

#### Ecology

Specimens were found on the trees or on the ground along the stream between 19:30 and 21:30 h. The surrounding habitat was mixed secondary forest of hardwoods and shrubs.

### Leptobrachella nahangensis

(Lathrop, Murphy, Orlov & Ho, 1998)

0A08F9B9-3ADB-5DFB-8AA1-21FE0EFB4F7C

#### Materials

- **Type status:** Other material. **Occurrence:** catalogNumber: IEBR A.4885; individualCount: 1; sex: male; lifeStage: adult; **Taxon:** scientificName: *Leptobrachellanahangensis*; class: Amphibia; order: Anura; family: Megophryidae; genus: Leptobrachella; specificEpithet: *nahangensis*; scientificNameAuthorship: Lathrop, Murphy, Orlov & Ho, 1998; **Location:** country: Vietnam; countryCode: VN; stateProvince: Bac Kan; locality: Nam Xuan Lac HSCA; verbatimElevation: 696 m; verbatimLatitude: 22°16.418’N; verbatimLongitude: 105°30.483’E; verbatimCoordinateSystem: WGS84; **Event:** eventDate: 25 August 2020; eventRemarks: collected by L. M. Anh, D. H. Quyen, and P. Q. Tien; **Record Level:** language: en; collectionCode: Amphibia; basisOfRecord: PreservedSpecimen
- **Type status:** Other material. **Occurrence:** catalogNumber: IEBR A.4886; individualCount: 1; sex: female; lifeStage: adult; **Taxon:** scientificName: *Leptobrachellanahangensis*; class: Amphibia; order: Anura; family: Megophryidae; genus: Leptobrachella; specificEpithet: *nahangensis*; scientificNameAuthorship: Lathrop, Murphy, Orlov & Ho, 1998; **Location:** country: Vietnam; countryCode: VN; stateProvince: Bac Kan; locality: Nam Xuan Lac HSCA; verbatimElevation: 696 m; verbatimLatitude: 22°17.260’N; verbatimLongitude: 105°31.138’E; verbatimCoordinateSystem: WGS84; **Event:** eventDate: 25 August 2020; eventRemarks: collected by L. M. Anh, D. H. Quyen, and P. Q. Tien; **Record Level:** language: en; collectionCode: Amphibia; basisOfRecord: PreservedSpecimen
- **Type status:** Other material. **Occurrence:** catalogNumber: IEBR A.4887; individualCount: 1; sex: male; lifeStage: adult; **Taxon:** scientificName: *Leptobrachellanahangensis*; class: Amphibia; order: Anura; family: Megophryidae; genus: Leptobrachella; specificEpithet: *nahangensis*; scientificNameAuthorship: Lathrop, Murphy, Orlov & Ho, 1998; **Location:** country: Vietnam; countryCode: VN; stateProvince: Bac Kan; locality: Nam Xuan Lac HSCA; verbatimElevation: 697 m; verbatimLatitude: 22°16.807’N; verbatimLongitude: 105°31.748’E; verbatimCoordinateSystem: WGS84; **Event:** eventDate: 26August 2020; eventRemarks: collected by L. M. Anh and D. H. Quyen; **Record Level:** language: en; collectionCode: Amphibia; basisOfRecord: PreservedSpecimen
- **Type status:** Other material. **Occurrence:** catalogNumber: IEBR A.4888; individualCount: 1; sex: female; lifeStage: adult; **Taxon:** scientificName: *Leptobrachellanahangensis*; class: Amphibia; order: Anura; family: Megophryidae; genus: Leptobrachella; specificEpithet: *nahangensis*; scientificNameAuthorship: Lathrop, Murphy, Orlov & Ho, 1998; **Location:** country: Vietnam; countryCode: VN; stateProvince: Bac Kan; locality: Nam Xuan Lac HSCA; verbatimElevation: 697 m; verbatimLatitude: 22°16.807’N; verbatimLongitude: 105°31.748’E; verbatimCoordinateSystem: WGS84; **Event:** eventDate: 26August 2020; eventRemarks: collected by L. M. Anh and D. H. Quyen; **Record Level:** language: en; collectionCode: Amphibia; basisOfRecord: PreservedSpecimen
- **Type status:** Other material. **Occurrence:** catalogNumber: IEBR A.4889; individualCount: 1; sex: male; lifeStage: adult; **Taxon:** scientificName: *Leptobrachellanahangensis*; class: Amphibia; order: Anura; family: Megophryidae; genus: Leptobrachella; specificEpithet: *nahangensis*; scientificNameAuthorship: Lathrop, Murphy, Orlov & Ho, 1998; **Location:** country: Vietnam; countryCode: VN; stateProvince: Bac Kan; locality: Nam Xuan Lac HSCA; verbatimElevation: 746 m; verbatimLatitude: 22°17.587’N; verbatimLongitude: 105°30.562’E; verbatimCoordinateSystem: WGS84; **Event:** eventDate: 24April 2021; eventRemarks: collected by H. V. Chung and P. Q. Tien; **Record Level:** language: en; collectionCode: Amphibia; basisOfRecord: PreservedSpecimen
- **Type status:** Other material. **Occurrence:** catalogNumber: IEBR A.4890; individualCount: 1; sex: female; lifeStage: adult; **Taxon:** scientificName: *Leptobrachellanahangensis*; class: Amphibia; order: Anura; family: Megophryidae; genus: Leptobrachella; specificEpithet: *nahangensis*; scientificNameAuthorship: Lathrop, Murphy, Orlov & Ho, 1998; **Location:** country: Vietnam; countryCode: VN; stateProvince: Bac Kan; locality: Nam Xuan Lac HSCA; verbatimElevation: 762 m; verbatimLatitude: 22°16’.372’N; verbatimLongitude: 105°31.150’E; verbatimCoordinateSystem: WGS84; **Event:** eventDate: 28August 2020; eventRemarks: collected by P. Q. Tien; **Record Level:** language: en; collectionCode: Amphibia; basisOfRecord: PreservedSpecimen

#### Description

Size medium (SVL 39.2-41.3 mm in males; SVL 48.3-54.6 mm in females); head longer than wide (HL 16.5-18.7 mm, HW 14.8-15.3 mm in males; HL 19.0-21.6 mm, HW 17.7-20.0 mm in females); snout round, longer than eye diameter (RL 6.1-6.6 mm, ED 4.9-5.6 mm in males; RL 7.4-8.1 mm, ED 5.9-7.2 mm in females); nostrils situated dorsolaterally on snout, closer to the tip of snout than to eye (NS 2.1-2.6 mm, EN 3.2-4.2 mm in males; NS 2.6-3.0 mm, EN 4.4-5.7 mm in females); canthus round, flat in lateral view, loreal region oblique and concave; tympanum distinct, round; vomerine teeth absent; tongue notched posteriorly. Forelimbs: Forearm slender (FLL 9.1-11.0 mm in males; FLL 12.8-14.0 mm in females), hand length (HAL 22.5-24.2 mm in males; HAL 28.2-30.1 mm in females); relative finger lengths I < II < IV < III; tips of fingers slightly swollen; fingers free of webbing. Hind-limbs: Thigh length (FeL 18.4-20.2 mm in males; FeL 24.4-25.1 mm in females); tibia six times longer than wide in males (TbL 19.0-20.8 mm, TbW 3.2-3.4 mm), tibia five times longer than wide in females (TbL 23.2-25.3 mm, TbW 3.6-5.4 mm); relative toe lengths I < II < V < III < IV; toes with rudimentary webbing; tibio-tarsal articulation reaching to the eye when leg adpressed along body. Skin: Dorsal surface head and body smooth with small pustules and minute tubercles uniformly distributed; supratympanic fold distinct; flanks smooth, large tubercles near waist absent; throat, chest, belly and ventral surface of thighs smooth.

**Figure 6.**
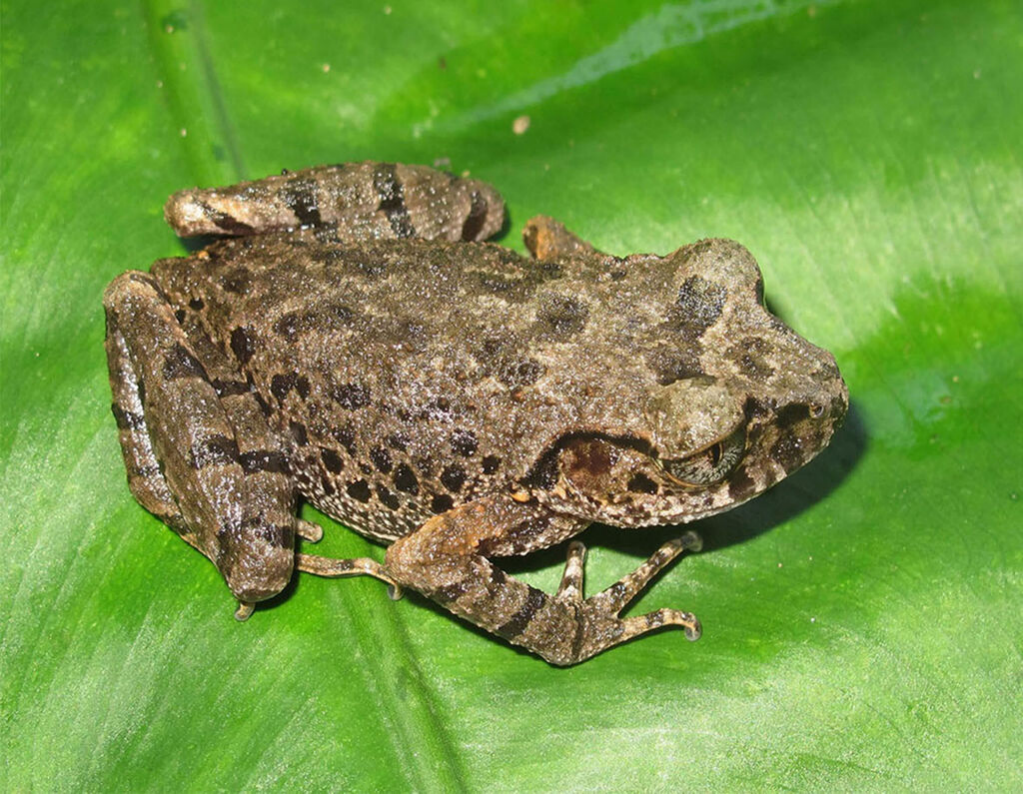
The male specimen of *Leptobrachellanahangensis* (IEBR A.4885; SVL 41.3 mm) in life.

Colouration in life: Dorsal surface of body and limbs grey; dorsum covered with irregular, diffuse dark grey and black spots; flanks light grey with a series of large well defined black spots; a pair of vertical bars on upper lip; limbs and digits with transverse dark bars. Forearm and heels yellow-orange; belly pinkish-white (Fig. 6) (determination after Lathrop et al. (1998)).

#### Distribution

In Vietnam, *L.nahangensis* was previously known only from Tuyen Quang Province (Nguyen et al. 2009, Frost 2021). The new record of the species from Bac Kan found approximately 19 km from the type locality in Tuyen Quang Province.

#### Ecology

Specimens were found on the trees along the stream, between 20:00 and 22:00 h. The surrounding habitat was mixed secondary forest of small hardwoods and shrubs.

### Quasipaa boulengeri

(Günther, 1889)

40D8B07F-C086-522D-ACEE-EE2D3C64F62B

#### Materials

- **Type status:** Other material. **Occurrence:** catalogNumber: IEBR A.4891; individualCount: 1; sex: female; lifeStage: adult; **Taxon:** scientificName: *Quasipaaboulengeri*; class: Amphibia; order: Anura; family: Dicroglossidae; genus: Quasipaa; specificEpithet: *boulengeri*; scientificNameAuthorship: Günther, 1889; **Location:** country: Vietnam; countryCode: VN; stateProvince: Bac Kan; locality: Nam Xuan Lac HSCA; verbatimElevation: 773 m; verbatimLatitude: 22°17.525’N; verbatimLongitude: 105°30.993’E; verbatimCoordinateSystem: WGS84; **Event:** eventDate: 27 August 2020; eventRemarks: collected by P. Q. Tien; **Record Level:** language: en; collectionCode: Amphibia; basisOfRecord: PreservedSpecimen
- **Type status:** Other material. **Occurrence:** catalogNumber: IEBR A.4892; individualCount: 1; sex: female; lifeStage: adult; **Taxon:** scientificName: *Quasipaaboulengeri*; class: Amphibia; order: Anura; family: Dicroglossidae; genus: Quasipaa; specificEpithet: *boulengeri*; scientificNameAuthorship: Günther, 1889; **Location:** country: Vietnam; countryCode: VN; stateProvince: Bac Kan; locality: Nam Xuan Lac HSCA; verbatimElevation: 762 m; verbatimLatitude: 22°16.372’N; verbatimLongitude: 105°31.150’E; verbatimCoordinateSystem: WGS84; **Event:** eventDate: 28August 2020; eventRemarks: collected by P. Q. Tien; **Record Level:** language: en; collectionCode: Amphibia; basisOfRecord: PreservedSpecimen

#### Description

Size large (SVL 100.4-106.5 mm); head shorter than wide (HL 42.8-45.2 mm, HW 45.1-45.3 mm); snout obtusely pointed in dorsal view, longer than eye diameter (RL 15.6 mm, ED 10.9-12.5 mm); nostrils lateral, round, closer to eye than the tip of snout (NS 7.5-8.0 mm, EN 7.3-7.4 mm); canthus rostralis short, but distinct, loreal region oblique, shallowly concave; tympanum distinct, round; vomerine teeth present; tongue deeped notched. Forelimbs: Forearm short (FLL 17.4-21.4 mm), hand length (HAL 48.2-48.4 mm); relative finger lengths I < II < IV < III, tips of fingers obtuse or slightly swollen; fingers free of webbing. Hind-limbs: Thigh short (FeL 51.8-55.2 mm); tibia three times longer than wide (TbL 54.4 mm, TbW 19.4-20.2 mm); relative toe lengths I < II < V < III < IV; toes fully webbed; tibio-tarsal articulation reaching to the eye when leg adpressed along body. Skin: Skin of the upper parts covered with large elongated warts and small oval tubercles; supratympanic fold distinct; flanks with small round tubercles, more dense near dorsolateral folds; belly smooth.

**Figure 7.**
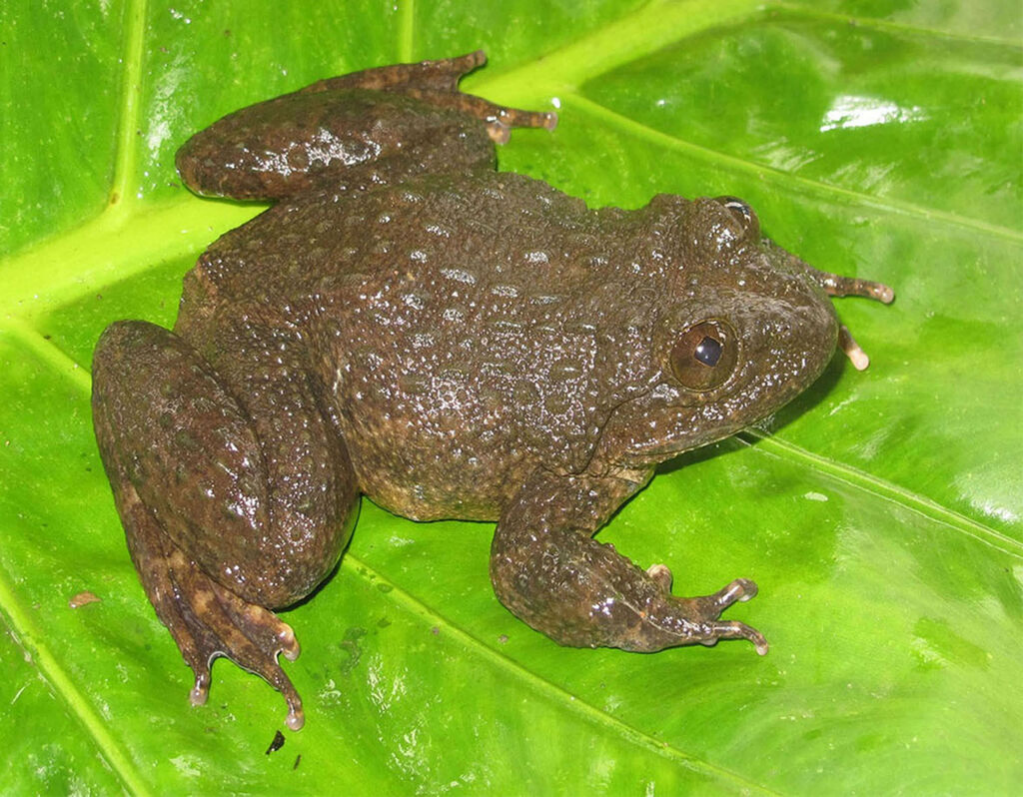
The female specimen of *Quasipaaboulengeri* (IEBR A.4891; SVL 106.5 mm) in life.

Colouration in life: Dorsal surface of head body and flank dark grey; dorsum with black large elongated warts; dorsal surface of limbs with dark cross bars; ventral surface cream (Fig. 7) (determination after Liu (1950)).

#### Distribution

In Vietnam, *Q.boulengeri* was known from Cao Bang, Son La, Tuyen Quang and Nghe An Provinces (Nguyen et al. 2009, Frost 2021). Elsewhere, this species has been recorded from China (Frost 2021).

#### Ecology

Specimens were found in the stream, at 20:30 h. The surrounding habitat was mixed secondary forest of medium hardwoods and shrubs.

### Odorrana lipuensis

Mo, Chen, Wu, Zhang & Zhou, 2015

D3976999-BA2F-564B-BC77-970FEDC5E633

#### Materials

- **Type status:** Other material. **Occurrence:** catalogNumber: IEBR A.4893; individualCount: 1; sex: male; lifeStage: adult; **Taxon:** scientificName: *Odorranalipuensis*; class: Amphibia; order: Anura; family: Ranidae; genus: Odorrana; specificEpithet: *lipuensis*; scientificNameAuthorship: Mo, Chen, Wu, Zhang & Zhou, 2015; **Location:** country: Vietnam; countryCode: VN; stateProvince: Bac Kan; locality: Nam Xuan Lac HSCA; verbatimElevation: 723 m; verbatimLatitude: 22°16.450’N; verbatimLongitude: 105°30.712’E; verbatimCoordinateSystem: WGS84; **Event:** eventDate: 25 August 2020; eventRemarks: collected by L. M. Anh, D. H. Quyen, and P. Q. Tien; **Record Level:** language: en; collectionCode: Amphibia; basisOfRecord: PreservedSpecimen
- **Type status:** Other material. **Occurrence:** catalogNumber: IEBR A.4894; individualCount: 1; sex: female; lifeStage: adult; **Taxon:** scientificNameID: *Odorranalipuensis*; scientificName: *Odorranalipuensis*; class: Amphibia; order: Anura; family: Ranidae; genus: Odorrana; specificEpithet: *lipuensis*; scientificNameAuthorship: Mo, Chen, Wu, Zhang & Zhou, 2015; **Location:** country: Vietnam; countryCode: VN; stateProvince: Bac Kan; locality: Nam Xuan Lac HSCA; verbatimElevation: 864 m; verbatimLatitude: 22°17.260’N; verbatimLongitude: 105°31.138’E; verbatimCoordinateSystem: WGS84; **Event:** eventDate: 24April 2021; eventRemarks: collected by H. V. Chung and P. Q. Tien; **Record Level:** language: en; collectionCode: Amphibia; basisOfRecord: PreservedSpecimen

#### Description

Size medium (SVL 45.2 mm in male; SVL 53.7 mm in female); head longer than wide (HL 17.1 mm, HW 14.8 mm in male; HL 21.4 mm, HW 18.6 mm in female); snout obtusely round in dorsal view, longer than eye diameter (RL 6.6 mm, ED 5.1 mm in male; RL 8.0 mm, ED 6.6 mm in female); nostrils round, closer to the tip of snout than to eye (NS 2.6 mm, EN 4.1 mm in male; NS 3.4 mm, EN 5.3 mm in female); canthus rostralis distinct, loreal region slightly concave and oblique; tympanum distinct round; vomerine teeth present; tongue deeply notched posteriorly. Forelimbs: Forearm slender (FLL 10.3 mm in male; FLL 11.8 mm in female), hand length (HAL 24.0 mm in male; HAL 30.1 mm in female); relative finger lengths I < II < IV < III, tips of fingers enlarged into discs; fingers free of webbing. Hind-limbs: Thigh slender (FeL 23.7 mm in male; FeL 29.7 mm in female); tibia five times longer than wide in the male (TbL 26.3 mm, TbW 4.6 mm), six times longer than wide in the female (TbL 32.8 mm, TbW 5.0 mm); relative toe lengths I < II < III < V < IV; webbing formula I½-½II0-1III0-1½IV1-0V; tibio-tarsal articulation reaching to the nostril when leg adpressed along body. Skin: Dorsal surface of head and body smooth; tiny spinules on flanks, upper edge of eyelid; anterior and posterior edge of tympanum; supratympanic fold indistinct, dorsolateral fold absent; throat, chest, belly and ventral surface of thigh smooth.

**Figure 8.**
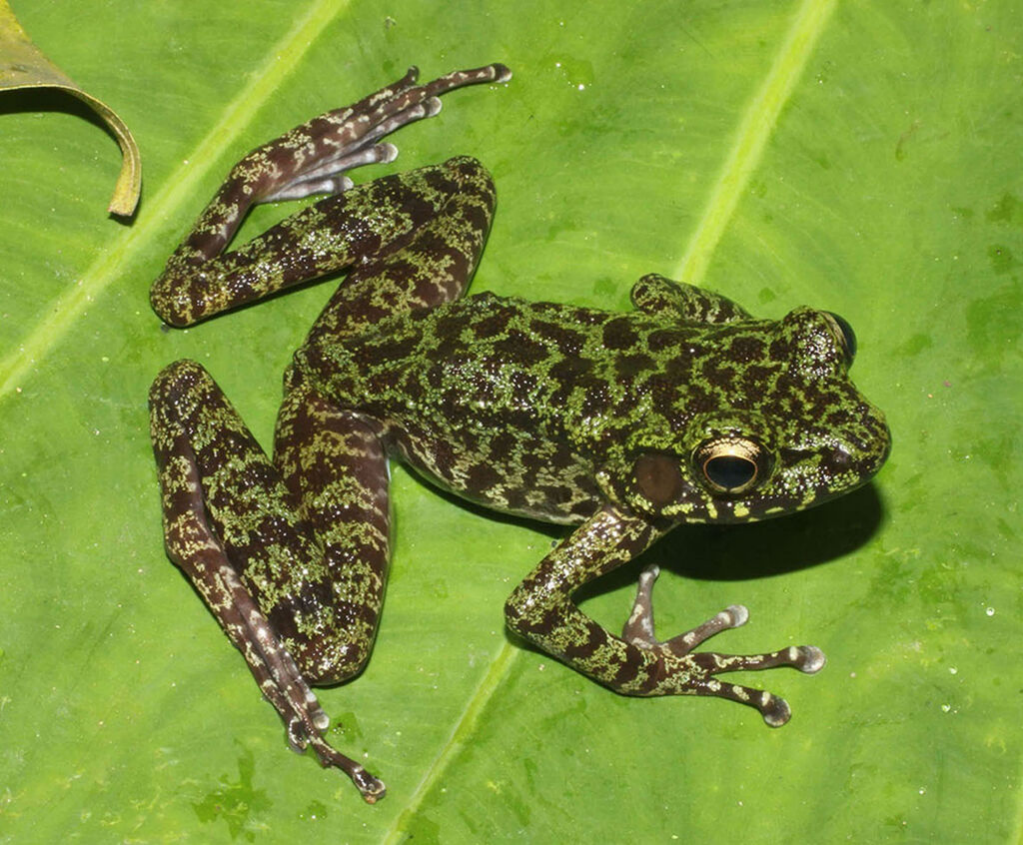
The male specimen of *Odorranalipuensis* (IEBR A.4893; SVL 45.2 mm) in life.

Colouration in life: Dorsum and upper part of flanks moss green with brown mottles, dorsal surface of limbs moss green with dark brown cross bars; upper lip with dark brown bars; throat, chest and belly cream with dark brown mottles (Fig. 8) (determination after Mo et al. (2015), Pham et al. (2016)).

#### Distribution

In Vietnam, *O.lipuensis* was known from Cao Bang and Tuyen Quang Provinces (Pham et al. 2016, Frost 2021). Elsewhere, this species has been recorded from China (Frost 2021).

#### Ecology

Specimens were found on trees, between 20:00 and 20:30 h, near the waterfall in a rocky stream. The surrounding habitat was mixed secondary forest of small hardwoods, shrubs and vines.

### Rana johnsi

Smith, 1921

6FB47F5F-A6ED-593C-9DD2-6C200DDDB95F

#### Materials

- **Type status:** Other material. **Occurrence:** catalogNumber: IEBR A.4895; individualCount: 1; sex: male; lifeStage: adult; **Taxon:** scientificName: *Ranajohnsi*; class: Amphibia; order: Anura; family: Ranidae; genus: Rana; specificEpithet: *johnsi*; scientificNameAuthorship: Smith, 1921; **Location:** country: Vietnam; countryCode: VN; stateProvince: Bac Kan; locality: Nam Xuan Lac HSCA; verbatimElevation: 714 m; verbatimLatitude: 22°16.492’N; verbatimLongitude: 105°31.165’E; verbatimCoordinateSystem: WGS84; **Event:** eventDate: 28August 2020; eventRemarks: collected by P. Q. Tien; **Record Level:** language: en; collectionCode: Amphibia; basisOfRecord: PreservedSpecimen
- **Type status:** Other material. **Occurrence:** catalogNumber: IEBR A.4896; individualCount: 1; sex: female; lifeStage: adult; **Taxon:** scientificName: *Ranajohnsi*; class: Amphibia; order: Anura; family: Ranidae; genus: Rana; specificEpithet: *johnsi*; scientificNameAuthorship: Smith, 1921; **Location:** country: Vietnam; countryCode: VN; stateProvince: Bac Kan; locality: Nam Xuan Lac HSCA; verbatimElevation: 696 m; verbatimLatitude: 22°16.418’N; verbatimLongitude: 105°30.650’E; verbatimCoordinateSystem: WGS84; **Event:** eventDate: 25 August 2020; eventRemarks: collected by L. M. Anh, D. H. Quyen and P. Q. Tien; **Record Level:** language: en; collectionCode: Amphibia; basisOfRecord: PreservedSpecimen
- **Type status:** Other material. **Occurrence:** catalogNumber: IEBR A.4897; individualCount: 1; sex: female; lifeStage: adult; **Taxon:** scientificName: *Ranajohnsi*; class: Amphibia; order: Anura; family: Ranidae; genus: Rana; specificEpithet: *johnsi*; scientificNameAuthorship: Smith, 1921; **Location:** country: Vietnam; countryCode: VN; stateProvince: Bac Kan; locality: Nam Xuan Lac HSCA; verbatimElevation: 696 m; verbatimLatitude: 22°16.418’N; verbatimLongitude: 105°30.650’E; verbatimCoordinateSystem: WGS84; **Event:** eventDate: 25August 2020; eventRemarks: collected by L. M. Anh, D. H. Quyen and P. Q. Tien; **Record Level:** language: en; collectionCode: Amphibia; basisOfRecord: PreservedSpecimen
- **Type status:** Other material. **Occurrence:** catalogNumber: IEBR A.4898; individualCount: 1; sex: female; lifeStage: adult; **Taxon:** scientificName: *Ranajohnsi*; class: Amphibia; order: Anura; family: Ranidae; genus: Rana; specificEpithet: *johnsi*; scientificNameAuthorship: Smith, 1921; **Location:** country: Vietnam; countryCode: VN; stateProvince: Bac Kan; locality: Nam Xuan Lac HSCA; verbatimElevation: 723 m; verbatimLatitude: 22°17.645’N; verbatimLongitude: 105°30.445’E; verbatimCoordinateSystem: WGS84; **Event:** eventDate: 27August 2020; eventRemarks: collected by P. Q. Tien; **Record Level:** language: en; collectionCode: Amphibia; basisOfRecord: PreservedSpecimen

#### Description

Size medium (SVL 39.7 mm in male; SVL 56.4-60.3 mm in females); head longer than wide (HL 14.5 mm, HW 11.2 mm in the male; HL 22.1-24.0 mm, HW 17.9-19.1 mm in females); snout obtusely pointed, longer than eye diameter (RL 5.9 mm, ED 4.6 mm in male; RL 8.6-9.0 mm, ED 6.2-6.6 mm in females); nostrils round, closer to the tip of snout than to eye (NS 2.4 mm, EN 3.9 mm in male; NS 3.4-4.2 mm, EN 5.1-5.3 mm in females); canthus rostralis distinct, loreal region concave; tympanum eliptic, distinct; vomerine teeth present; tongue notched posteriorly. Forelimbs: Forearm slender (FLL 8.9 mm in male; FLL 11.0-12.6 mm in females), hand length (HAL 18.7 mm in male; HAL 26.5-27.0 mm in females); relative finger lengths I < II < IV < III, tips of fingers not enlarged; fingers free of webbing. Hind-limbs: Thigh slender, long (FeL 21.6 mm in male; FeL 33.2-33.9 mm in females); tibia seven times longer than wide in male (TbL 25.3 mm, TbW 3.3 mm); tibia six times longer than wide in females (TbL 36.5-38.8 mm, TbW 6.1-7.2 mm); relative toe lengths I < II < III < V < IV; webbing formula I0-½II0-1III½-1IV1-0V; tibio-tarsal articulation reaching to the nostril when leg adpressed along body. Skin: Dorsal surface of head and body smooth with some small tubercles; supratympanic fold distinct, some short, oblique dermal folds on limbs; a Λ-shaped fold between shoulders; dorsolateral fold present; ventral surface smooth.

**Figure 9.**
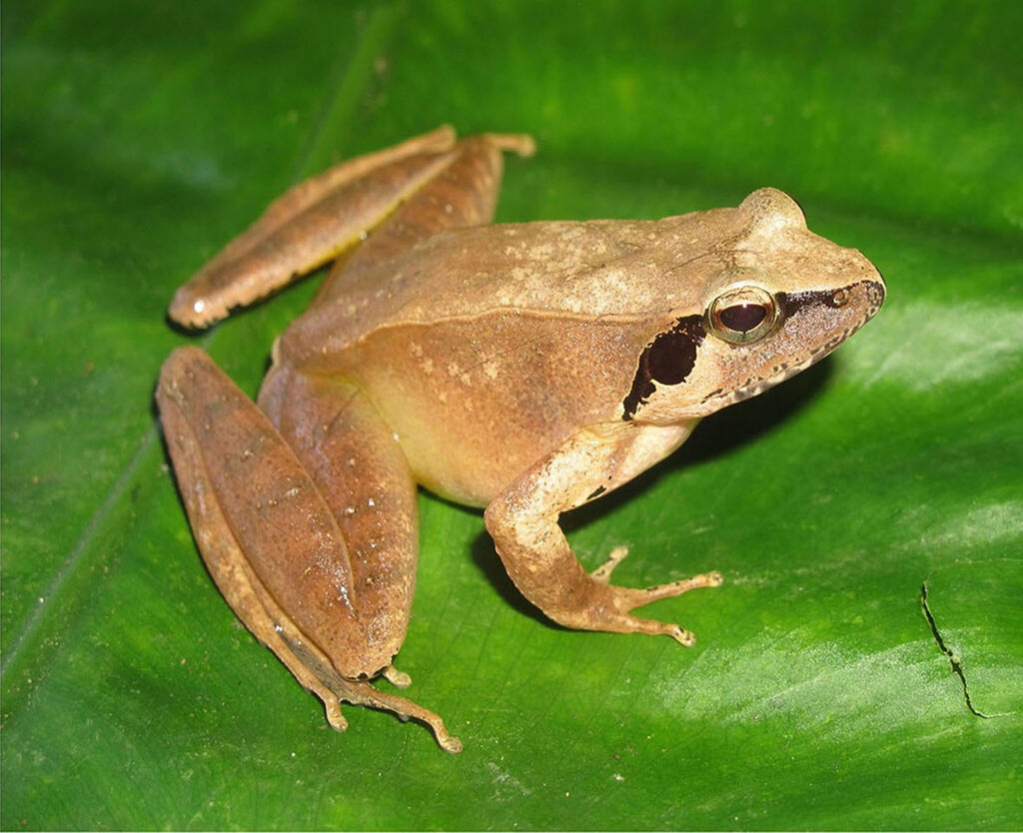
The male specimen of *Ranajohnsi* (IEBR A.4895; SVL 39.7 mm) in life.

Colouration in life: Dorsal surface light brown; flanks whitish-brown; dorsal surface of limbs with dark brown transverse bars; a small black stripe from nostril to eye; sides of limbs with dark pattern; ventral surface yellowish-white; gular region marbled with grey; ventral surface of hind-limbs yellow (Fig. 9) (determination after Bourret (1942), Inger et al. (1999)).

#### Distribution

In Vietnam, *R.johnsi* was known from Lao Cai and Ha Giang Provinces in the North to Lam Dong and Dong Nai Provinces (Nguyen et al. 2009, Frost 2021). Elsewhere, this species has been recorded from China, Taiwan, Thailand and Cambodia (Nguyen et al. 2009, Frost 2021).

#### Ecology

Specimens were found on the ground, between 19:00 and 20:30 h. The surrounding habitat was mixed secondary forest of small hardwoods and vines.

#### Notes

This species is morphologically similar to *Nidiranalini*. However, it differs from *N.lini* by having a smaller body size in the male (SVL 39.7 mm vs. 44.2-61.2 mm in *N.lini*) and the absence of vocal sacs in males (vs. present in *N.lini*) (Chou 1999).

### Gracixalus nonggangensis

Mo, Zhang, Luo, Zhou & Chen, 2013

3A811CDB-93C9-5C82-8567-022ECD3C65EC

#### Materials

- **Type status:** Other material. **Occurrence:** catalogNumber: IEBR A.4899; individualCount: 1; sex: male; lifeStage: adult; **Taxon:** scientificName: *Gracixalusnonggangensis*; class: Amphibia; order: Anura; family: Rhacophoridae; genus: Gracixalus; specificEpithet: *nonggangensis*; scientificNameAuthorship: Mo, Zhang, Luo, Zhou & Chen, 2013; **Location:** country: Vietnam; countryCode: VN; stateProvince: Bac Kan; locality: Nam Xuan Lac HSCA; verbatimElevation: 746 m; verbatimLatitude: 22°17.587’N; verbatimLongitude: 105°30.445’E; verbatimCoordinateSystem: WGS84; **Event:** eventDate: 24April 2021; eventRemarks: collected by H. V. Chung an P. Q. Tien; **Record Level:** language: en; collectionCode: Amphibia; basisOfRecord: PreservedSpecimen

#### Description

Size small (SVL 32.9 mm); head longer than wide (HL 12.0 mm, HW 11.8 mm); snout round, smaller than eye diameter (RL 4.6 mm, ED 4.9 mm); nostrils oval, closer to the tip of snout than to eye (NS 2.0 mm, EN 2.5 mm); canthal edges rounded, loreal region oblique, slightly concave; tympanum distinct, round; vomerine teeth absent; tongue notched posteriorly. Forelimbs: Forearm short and gracile (FLL 7.2 mm), hand length (HAL 16.3 mm); relative finger lengths I < II < IV < III, tips of fingers enlarged into discs; fingers free of webbing. Hinlimbs: Thigh moderately long (FeL 18.2 mm); tibia six times longer than wide (TbL 19.0 mm, TbW 2.9 mm); relative toe lengths I < II < III < V < IV; webbing formula I1-2II1-2III1-2IV2-1V; tibio-tarsal articulation reaching to the nostril when leg adpressed along body. Skin: Dorsum and dorsal parts of head; limbs overall smooth, but with some small tubercles; supratympanic fold indistinct; ventral part of forearm smooth; dorsolateral folds absent; throat, chest, belly and ventral part of thighs granular.

**Figure 10.**
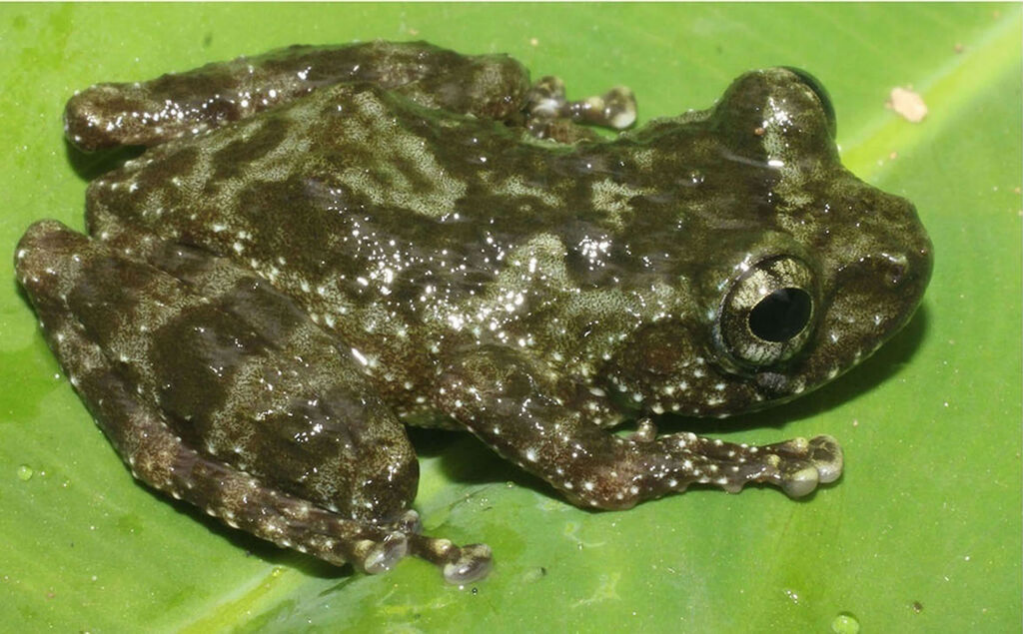
The male specimen of *Gracixalusnonggangensis* (IEBR A.4899; SVL 32.9 mm) in life.

Colouration in life: Dorsal surface of head and body, flank green olive, a dark green irregular patch running from between eyes to shoulder; upper and lower lip green olive with some creamy white spots; dorsal part of limbs green olive with transverse dark green bands; throat and margin of throat, chest and belly marbled with white; ventral part of limbs greyish-white (Fig. 10) (determination after Nguyen et al. (2013), Mo et al. (2013)).

#### Distribution

In Vietnam, *G.nonggangensis* was previously known from Cao Bang Province (Nguyen et al. 2013). Elsewhere, this species has been recorded from China (Frost 2021).

#### Ecology

Specimen was found at 20:00 h on a tree, about 0.5 m above the ground. The surrounding habitat was mixed secondary forest of hardwoods and shrubs.

#### Notes

The specimens of *G.nonggangensis* from Bac Kan only differ slightly from those in the description of Mo et al. (2013) in having a rostral length shorter than eye diameter (vs. rostral length longer than eye diameter).

### Rhacophorus orlovi

Ziegler & Köhler, 2001

B26E3373-E343-51EB-B513-C84201DE47AD

#### Materials

- **Type status:** Other material. **Occurrence:** catalogNumber: IEBR A.4900; individualCount: 1; sex: male; lifeStage: adult; **Taxon:** scientificName: *Rhacophorusorlovi*; class: Amphibia; order: Anura; family: Rhacophoridae; genus: Rhacophorus; specificEpithet: *orlovi*; scientificNameAuthorship: Ziegler & Köhler, 2001; **Location:** country: Vietnam; countryCode: VN; stateProvince: Bac Kan; locality: Nam Xuan Lac HSCA; verbatimElevation: 723 m; verbatimLatitude: 22°16.450’N; verbatimLongitude: 105°30.712’E; verbatimCoordinateSystem: WGS84; **Event:** eventDate: 25August 2020; eventRemarks: collected by L. M. Anh, D. H. Quyen, and P. Q. Tien; **Record Level:** language: en; collectionCode: Amphibia; basisOfRecord: PreservedSpecimen
- **Type status:** Other material. **Occurrence:** catalogNumber: IEBR A.4901; individualCount: 1; sex: male; lifeStage: adult; **Taxon:** scientificName: *Rhacophorusorlovi*; class: Amphibia; order: Anura; family: Rhacophoridae; genus: Rhacophorus; specificEpithet: *orlovi*; scientificNameAuthorship: Ziegler & Köhler, 2001; **Location:** country: Vietnam; countryCode: VN; stateProvince: Bac Kan; locality: Nam Xuan Lac HSCA; verbatimElevation: 723 m; verbatimLatitude: 22°16.450’N; verbatimLongitude: 105°30.712’E; verbatimCoordinateSystem: WGS84; **Event:** eventDate: 25August 2020; eventRemarks: collected by L. M. Anh, D. H. Quyen, and P. Q. Tien; **Record Level:** language: en; collectionCode: Amphibia; basisOfRecord: PreservedSpecimen
- **Type status:** Other material. **Occurrence:** catalogNumber: IEBR A.4902; individualCount: 1; sex: male; lifeStage: adult; **Taxon:** scientificName: *Rhacophorusorlovi*; class: Amphibia; order: Anura; family: Rhacophoridae; genus: Rhacophorus; specificEpithet: *orlovi*; scientificNameAuthorship: Ziegler & Köhler, 2001; **Location:** country: Vietnam; countryCode: VN; stateProvince: Bac Kan; locality: Nam Xuan Lac HSCA; verbatimElevation: 696 m; verbatimLatitude: 22°16.328’N; verbatimLongitude: 105°30.503’E; verbatimCoordinateSystem: WGS84; **Event:** eventDate: 24April 2021; eventRemarks: collected by L. M. Anh and D. H. Quyen; **Record Level:** language: en; collectionCode: Amphibia; basisOfRecord: PreservedSpecimen
- **Type status:** Other material. **Occurrence:** catalogNumber: IEBR A.4903; individualCount: 1; sex: female; lifeStage: adult; **Taxon:** scientificName: *Rhacophorusorlovi*; class: Amphibia; order: Anura; family: Rhacophoridae; genus: Rhacophorus; specificEpithet: *orlovi*; scientificNameAuthorship: Ziegler & Köhler, 2001; **Location:** country: Vietnam; countryCode: VN; stateProvince: Bac Kan; locality: Nam Xuan Lac HSCA; verbatimElevation: 696 m; verbatimLatitude: 22°16.328’N; verbatimLongitude: 105°30.503’E; verbatimCoordinateSystem: WGS84; **Event:** eventDate: 24 April 2021; eventRemarks: collected by L. M. Anh and D. H. Quyen; **Record Level:** language: en; collectionCode: Amphibia; basisOfRecord: PreservedSpecimen
- **Type status:** Other material. **Occurrence:** catalogNumber: IEBR A.4904; individualCount: 1; sex: male; lifeStage: adult; **Taxon:** scientificName: *Rhacophorusorlovi*; class: Amphibia; order: Anura; family: Rhacophoridae; genus: Rhacophorus; specificEpithet: *orlovi*; scientificNameAuthorship: Ziegler & Köhler, 2001; **Location:** country: Vietnam; countryCode: VN; stateProvince: Bac Kan; locality: Nam Xuan Lac HSCA; verbatimElevation: 762 m; verbatimLatitude: 22°16.372’N; verbatimLongitude: 105°31.150’E; verbatimCoordinateSystem: WGS84; **Event:** eventDate: 28August 2020; eventRemarks: collected by P. Q. Tien; **Record Level:** language: en; collectionCode: Amphibia; basisOfRecord: PreservedSpecimen

#### Description

Size medium (SVL 40.6-42.8 mm in males; SVL 48.7-57.6 mm in females); head longer than wide (HL 14.9-16.4 mm, HW 14.5-15.7 mm in males; HL 19.0-22.8 mm, HW 18.3-20.9 mm in females); snout slightly pointed, longer than eye diameter (RL 6.4-7.1 mm, ED 4.6-5.6 mm in males; RL 7.5-9.5 mm, ED 6.1-7.5 mm in females); nostrils oval, closer to the tip of snout than to eye (NS 32.4-3.0 mm, EN 4.1-4.7 mm in males; NS 3.6-3.8 mm, EN 2.3-6.1 mm in females); canthus rostralis well-developed, slightly constricted, loreal region concave; tympanum distinct, round; vomerine teeth absent; tongue deeply notched posteriorly. Forelimbs: Forearm slender (FLL 8.1-9.0 mm in males; FLL 12.0-12.4 mm in females), hand length (HAL 21.1-21.7 mm in males; HAL 26.8-32.2 mm in females); relative finger lengths I < II < IV < III, tips of fingers enlarged into discs; webbing formula I1-1½II1-2III1-1IV. Hind-limbs: Thigh slender (FeL 20.8-21.7 mm in males; FeL 26.5-30.3 mm in females); tibia six times longer than wide (TbL 22.3-25.1 mm, TbW 3.4-3.7 mm in males; TbL 27.8-31.4 mm, TbW 4.4-5.2 mm in females); relative toe lengths I < II < III < V < IV; webbing formula I½-1II⅓-1III½-1IV1-⅓V; tibio-tarsal articulation reaching to the position between the eye and nostril when leg adpressed along body. Skin: Dorsal surface of head, body and upper part of flanks smooth; supratympanic fold distinct; limbs without distinct dermal flaps and folds, except for a weakly developed fold along outer edge of 4^th^ finger and 5^th^ toe; weak tubercles and protuberances on outer edge of tarsus; throat, chest smooth; belly, ventral surface of limbs granular.

**Figure 11.**
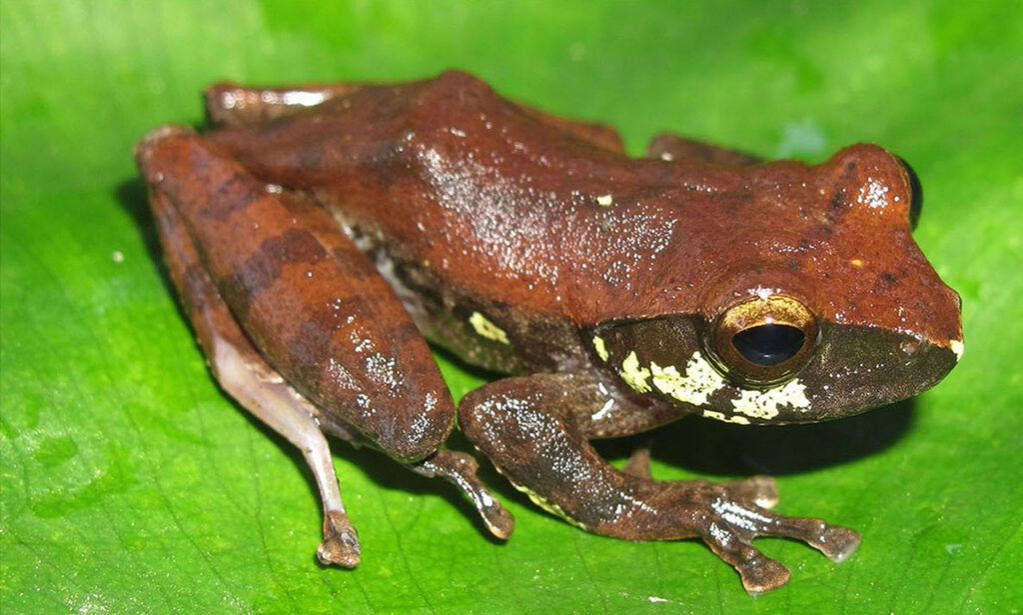
The male specimen of *Rhacophorusorlovi* (IEBR A.4900; SVL 40.6 mm) in life.

Colouration in life: Dorsal surface of head and body reddish-brown with some darker markings; flanks light brown with dark brown reticulation and yellow spots; ventral light grey with some indistinct small dark spots; limbs dorsally light reddish-brown with dark crossbars (Fig. 11) (determination after Ziegler and Köhler (2001), Ostroshabov et al. (2013)).

#### Distribution

In Vietnam, *R.orlovi* was known from Dien Bien and Son La Provinces in the North to Gia Lai Province in the Central Highlands (Nguyen et al. 2009). Elsewhere, this species has been recorded from Laos (Frost 2021).

#### Ecology

Specimens were found on trees along the trail, between 20:00 and 21:00 h, 2-3 m from the ground. The surrounding habitat consisted of mixed secondary forest composed of small hardwoods and vines.

## Discussion

Our new findings bring the total number of amphibian species to 43 in Bac Kan Province (Nguyen et al. 2009). Le et al. (2004) provided a list of the herpetofauna of Ba Be National Park with 16 recorded species of amphibian. Pham et al. (2015) report six new species of amphibians from Kim Hy Nature Reserve. These authors also recorded *Rhacophorusviridimaculatus*, a species originally described by Ostroshabov et al. (2013) from Ha Giang Province, for the first time from Bac Kan Province. However, the specimens from Nam Xuan Lac HSCA differ from those of *R.viridimaculatus* by having a smaller interorbital distance in males (IOD 5.1-5.6 mm vs. 8.76 mm in *R.viridimaculatus*) and the absence vocal sacs in males (vs. present in *R.viridimaculatus*) (Ostroshabov et al. 2013). Further phylogenetic studies will help to elucidate the taxonomic position of these morphologically-similar species. The amphibian fauna of Nam Xuan Lac HSCA, Bac Kan Province contains two species of conservation concern: one species endemic to Vietnam (*Leptobrachellanahangensis*) and one species (*Quasipaaboulengeri*) listed as Endangered by IUCN (2021). Additional surveys are required to obtain further data on the actual herpetofaunal diversity of Nam Xuan Lac Habitat and Species Conservation Area, as well as of Bac Kan Province.

## Supplementary Material

XML Treatment for Microhyla butleri

XML Treatment for Leptobrachella minima

XML Treatment for Leptobrachella nahangensis

XML Treatment for Quasipaa boulengeri

XML Treatment for Odorrana lipuensis

XML Treatment for Rana johnsi

XML Treatment for Gracixalus nonggangensis

XML Treatment for Rhacophorus orlovi
